# Invasive Carcinoma Ex-Pleomorphic Adenoma of the Lacrimal Gland with a Cystadenocarcinoma Component: A Case Report and Review of the Literature

**DOI:** 10.1155/2020/6482837

**Published:** 2020-08-12

**Authors:** Vamsee K. Neerukonda, Bryant Carruth, Maria Del Valle Estopinal

**Affiliations:** ^1^Department of Ophthalmology, State University of New York Upstate Medical University, Syracuse, New York, USA; ^2^Eye Plastic and Reconstructive Surgery of Central New York, 3400 Vickery Rd, North Syracuse, NY 13212, USA

## Abstract

Lacrimal gland neoplasms comprise up to 18% of all orbital masses clinically and histologically. Much of our current core knowledge regarding lacrimal gland tumors stems from prior study of their more common counterparts, the salivary glands. The prognosis for each lacrimal gland tumor is contingent upon proper clinical evaluation and ultimately the histopathologic diagnosis. We describe a case of an invasive carcinoma ex-pleomorphic adenoma (Ca-ex-PA) with a cystadenocarcinoma component arising from the lacrimal gland in the absence of any previously diagnosed pleomorphic adenoma (benign mixed tumor) or prior incisional surgery. This case illustrates the importance of the histopathologic assessment including immunohistochemistry and genetic testing to narrow a differential diagnosis and potentially aid or guide therapy in the future. Our finding suggests that carcinoma of the lacrimal gland may be derived from previously undiagnosed and perhaps even subclinical pleomorphic adenoma.

## 1. Introduction

Malignant mixed tumor of the lacrimal gland, also known as carcinoma ex-pleomorphic adenoma (Ca-ex-PA), is the third most frequent epithelial lacrimal gland tumor, after pleomorphic adenoma (PA) and adenoid cystic carcinoma (ACA) [1]. Clinically, affected patients tend to be 5 to 12 years older than those with PA and often present with an insidiously progressive mass of a lacrimal fossa that suddenly becomes symptomatic. Other reported clinical settings are patients without any previously known lacrimal gland tumor who develop a rapidly growing mass associated with pain and patients with one or more recurrences of PA who undergo malignant transformation [2]. Diagnostic imaging is critical for the clinical diagnosis of a lacrimal gland neoplasm. Computed tomography (CT) and magnetic resonance imaging (MRI) are ideal, but MRI remains the preferred method to visualize surrounding tissue and detect radiographic features concerning aggressive malignancy [3]. The incidence of a malignant transformation of PA increases with the duration of the tumor. Prior studies report that approximately less than 10% of PA undergoes malignant change within 20 years after the first treatment and 20% by the end of 30 years [3]. Ca-ex-PA can arise in patients without a known preexisting lacrimal gland tumor. The malignant component is most often adenocarcinoma, not otherwise specified; however, other histologic subtypes have been described such as adenoid cystic carcinoma, ductal carcinoma, mucoepidermoid carcinoma, and squamous cell carcinoma.

Herein, we report a case of an invasive Ca-ex-PA with an epithelial component consistent with a cystadenocarcinoma in the absence of any previously diagnosed PA and supported by immunohistochemical and molecular studies.

## 2. Case Presentation

**Figure 1:**
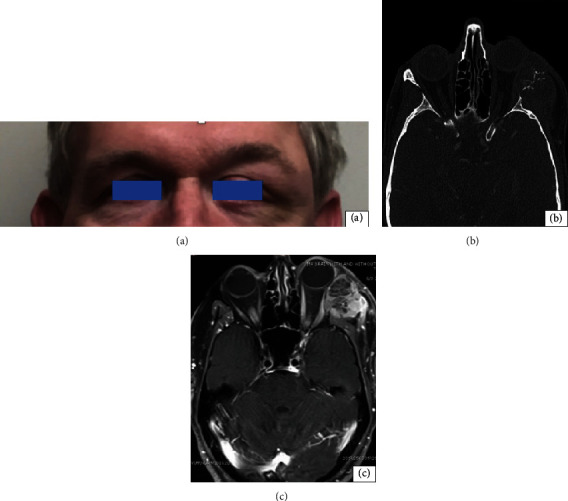
(a) Clinical photo of the patient at time of presentation, direct view. (b) Computed tomography maxillofacial, axial cut. Illustrating a 3.8 cm mass of the lateral orbit eroding the lateral orbital wall with a soft tissue component extending into the orbit causing proptosis and nonaxial displacement of the globe. (c) MRI brain and orbit T1 fat-suppressed image with gadolinium showing a lobulated mass with mixed cystic and solid components and inhomogeneous enhancement, involving the left lateral orbital wall and suprazygomatic left masticator space. Additionally, the mass demonstrates edema and enhancement suggesting infiltration by the mass.

A 64-year-old male patient presented to the emergency department due to left lateral canthal pain, tearing, and ipsilateral hearing loss over four months. The initial examination revealed a visual acuity of 20/20 bilaterally. The pupils were reactive without an afferent pupillary defect. Intraocular pressures were 14 and 15 mmHg, respectively. Confrontational visual fields and color plates were unremarkable. There was a complete abduction restriction of the left eye. The external examination revealed edematous upper and lower eyelids, predominantly overlying the lateral orbital rim associated with temporal sloping and a nontender, palpable, and fixed mass of the temporal fossa (Figure 1(a)). There was ptosis of the left upper eyelid with a reduced marginal reflex distance of two and a half millimeters (mm). Exophthalmometry measured 18 mm and 22 mm, with a base measurement of 100 mm. The anterior segment was otherwise normal. The fundus exam revealed symmetrically sharp and pink disc margins without pallor or edema.

**Figure 2:**
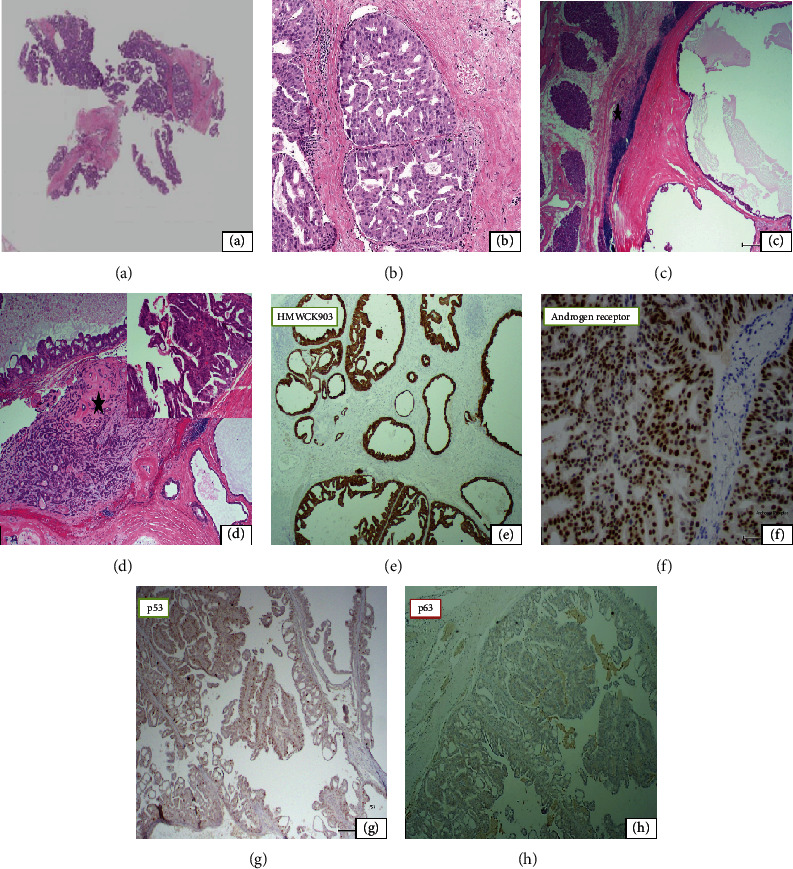
(a) Moderately differentiated adenocarcinoma involving cores of fibrous connective tissue. H&E 2x. (b) High power view of the tumor revealed a cribriform pattern, moderate nuclear pleomorphism, and scattered mitoses. H&E 40x. (c) Neoplastic cystic structures in close proximity to lacrimal gland tissue (star) in a hyalinized stroma and lymphoid proliferation. H&E 2x. (d) Tumor that arose from a focus of pleomorphic adenoma (star) depicting cystic structures with small papillae. H&E 4x. Inset: papillary projections lined by large cuboidal cells with moderate nuclear atypia and apocrine features. H&E 20x. (e) Tumor cells were strongly positive for HMWK903 (4x). (f) Nuclear expression of androgen receptor was present in the tumor (20x). (g) p53, positive in less than 50% of tumor cells (4x). (h) p63 was negative within the invasive component of the tumor (4x).

Maxillofacial CT scan with contrast showed a lytic lesion of the left orbital wall with associated heterogeneous soft tissue mass measuring 3.8 × 2.7 cm medially displacing the left lateral rectus muscle (Figure 1(b)). Magnetic resonance imaging of the brain and orbits revealed an enhancing infiltrating mass of the left lateral orbital wall extending into the left supra zygomatic masticator space (Figure 1(c)). A core guided needle biopsy was performed, and the hematoxylin-eosin- (H&E-) stained slide revealed a moderately differentiated adenocarcinoma involving fibrous connective tissue and demonstrating a cribriform architectural pattern with moderate cytologic atypia and individual cell necrosis (Figures 2(a) and 2(b)).

Positron emission tomography and CT of the chest, abdomen, and pelvis did not reveal any underlying malignancy or evidence of metastases. Subsequently, the patient underwent left orbital exenteration with eyelid sparing. Grossly, the specimen included orbital contents, frontal bone, portions of the frontal sinus, and zygomatic bone. Serial sectioning revealed a 3.5 × 2.5 cm multilocular cystic mass involving the lacrimal gland fossa abutting the globe superotemporally.

Histopathologically, the H&E-stained sections disclosed predominantly neoplastic cystic structures in the proximity of the lacrimal gland acini measuring 1 to 10 mm in diameter, infiltrating fibrous connective tissue and bone (Figure 2(c)). A small focus of pleomorphic adenoma was identified associated with a low-grade ductal carcinoma *in situ*. The invasive cystic component revealed intraluminal papillary architecture and cribriform arch formations of the lining epithelium (Figure 2(d)). The neoplastic epithelium was composed of medium- to large-sized cuboidal cells with intercalated duct-cell appearance, eosinophilic cytoplasm, and apocrine features. Small foci of invasive solid components were observed demonstrating cribriform architecture, moderate to severe nuclear pleomorphism, and up to 8 mitotic figures per high power field. Columnar cells with pseudostratified nuclei were also present with moderate nuclear atypia. Foci of ruptured cysts with hemorrhage, granulation tissue, lymphocytic infiltrate, and dystrophic calcification were also seen. No lymphovascular or perineural invasion was identified.

Immunohistochemical studies using monoclonal antibodies were performed with appropriate positive controls, encompassing Gata-3 (mouse monoclonal antibody; 1 : 100–1 : 500, predilute; Cell Marque, Rocklin, CA), gross cystic disease fluid protein 15 (GCDFP-15) (mouse monoclonal antibody; 1 : 30-1 : 60, dilute; Thermo Fisher Scientific, Fremont, CA), androgen receptor (AR) (rabbit monoclonal antibody; 1 : 50-1 : 200, dilute; Cell Marque, Rocklin, CA), SOX-10 (rabbit polyclonal antibody; 1 : 25-1 : 100, dilute; Cell Marque, Rocklin, CA), prostatic specific antigen (PSA) (mouse monoclonal antibody; 1 : 50-1 : 200, predilute; Cell Marque, Rocklin, CA), thyroid transcription factor 1 (TTF-1) (mouse monoclonal antibody; predilute at 7 micrograms; Ventana Medical Services, Tucson, AZ), p63 (mouse monoclonal antibody; predilute 1 : 100–1 : 200; Biocare Medical, Concord, CA), p53 (mouse monoclonal antibody; predilute at 2.5 micrograms; Ventana Medical Services, Tucson, AZ), high molecular weight cytokeratin 903 (HMWK903) (mouse monoclonal antibody; predilute 1 : 100-1 : 500; Cell Marque, Rocklin, CA), CAM 5.2 (mouse monoclonal antibody; predilute; Ventana Medical Services, Tucson, AZ), estrogen receptor (ER) (mouse monoclonal antibody; diluted 1 : 40-1 : 60; Vector Laboratories, Burlingame, CA), progesterone receptor (PR) (mouse monoclonal antibody; prediluted; Leica Biosystems, Newcastle, UK), Her2-neu (rabbit monoclonal antibody; predilute; Ventana Medical Services, Tucson, AZ), and Ki-67 (rabbit monoclonal antibody; predilute; Ventana Medical Services, Tucson, AZ). Immunohistochemical studies were performed on an automated stainer (Leica Biosystems, Inc., BOND III System, Buffalo Grove, IL). All antibodies and testing were performed in a CLIA-certified laboratory.

The invasive component of the tumor was positive for Gata-3, AR, HMWK903, and CAM5.2 and focally positive for GCDFP15 while negative for p63, ER, PR, SOX-10, PSA, and TTF-1 stains (Figures 2(e)–2(h)). p53 was positive in less than 50% of the tumor cells. The Ki-67 proliferative index of the tumor cells was low (5-10%). p63 and SOX-10 stains highlighted the PA and the *in situ* component of the tumor.

Detection of HER2 status by immunohistochemistry was equivocal (2+), and reflex testing using fluorescence in situ hybridization (FISH) was negative for HER2/neu gene amplification based on the updated 2018 guidelines of the American Society of Clinical Oncology/College of American Pathologists criteria for HER2/neu testing in breast cancer.

Molecular profiling using a next-generation sequencing- (NGS-) based assay (Foundation One) was performed on the lacrimal gland tumor (paraffin-embedded tissue) to identify genomic alterations within 315 cancer-related genes. The following genomic alterations were detected: *HRAS* (G13R), *PIK3CA* (E542K), and *BCORL1* (H215fs∗38); Microsatellite status MD-stable and tumor mutation burden (TMB) low; 1Muts/Mb.

The combined histopathologic, immunohistochemical, and molecular findings supported the diagnosis of invasive Ca-ex-PA disclosing a carcinomatous component with a predominant cystic growth pattern and focal solid areas reminiscent of a cystadenocarcinoma. The surgical margins were negative. In addition, the microscopic examination of the left eye globe revealed pseudoexfoliation syndrome and Fuchs' endothelial dystrophy.

The patient underwent postoperative adjuvant chemoradiation, followed by excision of the eyelids with no residual tumor. Follow-up examination showed no evidence of recurrence or metastatic disease nine months after completing adjuvant therapy.

## 3. Discussion

Much remains unknown about both the natural histologic progression and malignant transformation of Ca-ex-PA. We know that lacrimal gland neoplasms comprise 9-18% of orbital masses clinically [4, 5] and 5-18% of orbital masses histologically [1, 6–13], and nearly half of epithelial tumors are malignant [6]. Although uncommon, malignant transformation of PA can occur with incomplete excision. Invasive Ca-ex-PA can represent malignant transformation of PA, many of which develop recurrence or distant metastasis to the lung, bone, abdomen, and CNS [14]. This case highlights the importance of an immunohistochemical and genetic evaluation in complex lacrimal tumors.

The malignant epithelial component of Ca-ex-PA has morphological varieties including adenocarcinoma, adenoid cystic carcinoma, squamous cell carcinoma, mucoepidermoid carcinoma, and ductal carcinoma. It could be likely a mixture of subtypes [15]. Rarely, the only evidence of pleomorphic adenoma is the presence of large areas of hyalinized stroma composed of myoepithelial cells and a few ductal structures.

In the current case, the carcinomatous component of the tumor discloses predominantly an infiltrative cystic growth pattern reminiscent of a cystadenocarcinoma, previously described in neoplasms of the salivary and lacrimal glands. The term cystadenocarcinoma of the salivary gland has evolved. It encompasses a variety of tumors depicting a cystic pattern of growth to a subset of papillary and cystic malignant neoplasms that have indolent behavior. This is also observed in other low-grade salivary gland carcinomas; however, it is important to note that they can demonstrate infiltrative growth, local recurrence and metastasize to regional lymph nodes at the time of diagnosis.

Previously, Foss et al. [13] in a review of 57 cases of cystadenocarcinoma of the salivary gland used the following diagnostic criteria: (1) occurrence within a salivary gland, (2) invasive growth, (3) a predominantly cystic pattern of growth with or without a papillary component, and (4) the absence of acinar or mucoepidermoid differentiation or evidence of origin in a PA. In the same review, the predominant cell type varies among tumors and includes small cuboidal intercalated duct-like cells, large cuboidal cells, and tall columnar cells. The subgroups of the large cuboidal cells have central nuclei, abundant eosinophilic cytoplasm, large nucleoli, and apocrine features which are similar to those observed in this case. The fourth group has a combination of cell types. Furthermore, Foss et al. suggest that tumors with predominantly columnar cells are associated with increased metastatic potential.

The cystic growth pattern characteristic of this tumor is often associated with an inflammatory response due to ruptures of the dilated structures as noted in this case. Cyst formation in neoplasias of the salivary and lacrimal glands can behave as a mimicker of an inflammatory process. Pakdel et al. report a case of a spontaneous rupture of a PA masquerading as orbital cellulitis [16]. Histologically, a ruptured cystic space of a PA surrounded by a monocytic infiltrate and foreign body-type granulomas is described. In this paper, the authors consider the spontaneous rupture of the cystic space as an underlying mechanism for the acute presentation of this tumor.

Cystadenocarcinoma can have a broad differential diagnosis. The principal considerations include papillary cystic acinic cell adenocarcinoma, secretory carcinoma, mucoepidermoid carcinoma (MEC), and ductal carcinoma. Acinic cell carcinoma and secretory carcinoma are typically indolent, monotypic tumors that can disclose a papillary cystic architecture. Histologically, secretory carcinoma shares nearly identical growth patterns to acinic cell carcinoma but instead shows a multivacuolated eosinophilic cytoplasm, often with luminal and intracytoplasmic mucin and no true zymogen granules. Immunohistochemically, secretory carcinoma is S-100 and mammaglobin-positive and typically negative for DOG1 while acinic cell carcinoma shows the opposite staining profile. Additionally, secretory carcinoma harbors t(12;15)(p13;q25) resulting in an *ETV6-NTRK3* gene fusion [17]. MEC is usually composed of a mixture of predominantly epidermoid (squamoid) cells, abundant intermediate cells ranging from small basal cells with basophilic cytoplasm to larger cells with eosinophilic cytoplasm, and mucous cells. Well-differentiated MEC is a circumscribed tumor that can disclose glandular and cystic structures, lined by a single layer of mucus-secreting cells; however, the intermediate and high-grade tumors show solid nests or sheets of cells composed of primarily epidermoid cells with a scant cystic component and obvious invasion, severe pleomorphism, necrosis, and increased mitoses.

On the other hand, primary ductal adenocarcinoma of the salivary gland (SDC) originates from the excretory portion of the salivary duct, is a rare aggressive malignant epithelial tumor, and accounts for only 2% of epithelial lacrimal gland tumors. Histologically, the tumor is highly infiltrative and usually solid with occasional cystic areas disclosing breast-like ductal carcinoma features with central necrosis. Occasionally, cystadenocarcinoma with large cuboidal cells, eosinophilic cytoplasm, and high-grade nuclear atypia bears some similarities to ductal adenocarcinoma. However, papillary-cystic invasive growth is not usually seen in ductal adenocarcinoma [12]. The invasive component features trabeculae, ducts, and sheets of neoplastic cells in a desmoplastic stroma with perineural and vascular invasion. The latter is not commonly observed in cystadenocarcinoma. Immunohistochemically, the tumor cells are positive for a low molecular weight cytokeratin (CAM5.2), CK7, CEA, EMA, and GCDFP-15. Other than epithelial markers, this tumor expresses AR in up to 83% of invasive cases, HER2 positivity in 15-40% of cases, and p53 overexpression in all reported cases. The Ki-67 proliferative index is over 25%. The tumor is ER and PR negative.

Furthermore, in this case, the tumor also demonstrates cytomorphological features similar to that of a low- to intermediate-grade ductal carcinoma *in situ* of the breast. The latter findings might correlate with the previously described low-grade cribriform cystadenocarcinoma of the salivary gland, also known as low-grade salivary ductal carcinoma and salivary ductal carcinoma *in situ*, currently categorized as intraductal carcinoma, low grade, and high grade, respectively. These types of tumors show a variety of growth patterns both solid and cystic, ranging from cribriform to solid to micropapillary and are reminiscent of low-grade ductal carcinoma in situ of the breast. Focal infiltration may be noted [17].

The exact pathogenesis of Ca-ex-PA remains controversial. Prior studies have demonstrated that PA and Ca-ex-PA of the salivary and lacrimal glands share similar genomic profiles and frequently overexpress the PLAG1 oncoprotein [3, 18]. Harrison et al. [3], in their review, indicate that the development of Ca-ex-PA follows a multistep model of carcinogenesis with the progressive loss of heterozygosity at 8q, then 12q and finally 17p. Alterations including amplification, gene fusion, and translocations in 12q genes such as *HMGIC*, *HMGA2*, and *MDM2* may play a role in the malignant transformation. In the same study, the authors also mention that loss of 17p is usually common in Ca-ex-PA indicating tumor suppressor gene *p53* loss as this tumor evolves. Additionally, it appears that Ca-ex-PA and other malignant epithelial tumors, other than ACC, do not harbor *MYB* gene rearrangements or fusions. It is important to note that there does not appear to be a correlation between rearrangement status and clinical outcome [19].

Additional mutational analysis of the lacrimal gland carcinomas has been also evaluated [3], demonstrating that *RAS* (*KRAS*, *NRAS*), *PIK3CA*, and *MET* mutations are frequent in diverse epithelial neoplasms of the lacrimal gland with the highest proportion of mutations found in adenoid cystic carcinoma. *PIK3CA* and *MET* mutations can coexist with *RAS* mutations.

*PIK3CA* and *HRAS* mutations are detected in this case which correlates with alterations already described in lacrimal gland carcinomas; however, alterations in *MDM2*, *HMGA2*, *NTRK3*, *p53*, *PLAG1*, and *ETV6* among others were not observed.

In summary, this case demonstrates an invasive Ca-ex-PA with a malignant epithelial component that resembles the cystadenocarcinoma mixed cell type described by Foss et al. [13]. Immunohistochemically, the tumor was AR-positive while negative for ER, PR, and Her-2 with expression of p53 in less than 50% of the tumor cells. The prevalence of AR varies between the different subtypes of salivary gland carcinomas (SGC). In recent medical literature data, AR expression has been detected in as many as 98% of SDC [20]. Dalin et al. [20], in the same study, also indicate that adenocarcinoma and acinic cell carcinoma of the salivary gland express AR in 26% and 15% of the cases, respectively.

The current classification of the salivary gland tumors [17] and the combined histopathologic and immunohistochemical features suggest that this tumor could represent the results of the natural course of a low-grade cystadenocarcinoma with focal transformation into an intermediate/high-grade invasive ductal carcinoma ex-pleomorphic adenoma.

Our findings emphasize the importance of further exploration of Ca-ex-PA pathogenesis, especially the extent of disease and the histologic subtype. In addition, the immunohistochemical and genetic testing provides important support for the diagnosis as well as potentially guides future therapy and prognostic evaluation. Correctly identifying the type of malignant epithelial component is a significant factor in the survival of affected patients. To our knowledge, this patient represents the first case in which Ca-ex-PA of the lacrimal gland reveals an indolent epithelial malignancy with a low proliferative index, arising from an undiagnosed PA after many likely consecutive molecular alterations.

Lastly, due to the rarity of these tumors arising from the lacrimal gland, further studies are necessary to evaluate their biologic behavior and determine any correlation between Ca-ex-PA and pseudoexfoliation syndrome.
